# Associations of minority stress and employment discrimination with job quality among sexual- and gender-minority workers

**DOI:** 10.5271/sjweh.4221

**Published:** 2025-05-01

**Authors:** David J Kinitz, Nguyen K Tran, Faraz Vahid Shahidi, Joelle T Maslak, Annesa Flentje, Micah E Lubensky, Juno Obedin-Maliver, Mitchell R Lunn

**Affiliations:** 1 The PRIDE Study/PRIDEnet, Stanford University School of Medicine, Palo Alto, CA, USA.; 2 Division of Nephrology, Department of Medicine, Stanford University School of Medicine, Stanford, CA, USA.; 3 Department of Obstetrics and Gynecology, Stanford University School of Medicine, Stanford, CA, USA.; 4 Institute for Work & Health, Toronto, ON, Canada.; 5 Dalla Lana School of Public Health, University of Toronto, Toronto, ON, Canada.; 6 Gender Institute of Teaching and Advocacy, Metropolitan State University of Denver, Denver, CO, USA.; 7 Department of Community Health Systems, University of California San Francisco, San Francisco, CA, USA.; 8 Department of Psychiatry and Behavioral Sciences, University of California San Francisco, San Francisco CA, USA.; 9 Department of Epidemiology and Population Health, Stanford University School of Medicine, Stanford, CA, USA.

**Keywords:** cisheteronormativity, gender-based analysis, LGBTQ+, precarious employment, unemployment

## Abstract

**Objectives:**

This study aimed to describe how minority stress and employment discrimination are associated with job quality (*ie*, employment type and income) among sexual- and gender-minority (SGM) workers.

**Methods:**

We conducted a pooled cross-sectional analysis of the 2021 and 2022 surveys from a national study of SGM adults in the United States. Using multinomial logistic regression models and stratification by six gender groups, we examined associations of minority stress and past-year employment discrimination with job quality.

**Results:**

Among 4221 workers, 22.0% experienced precarious employment and 6.8% were unemployed. Approximately half earned ≤US$50 000. The mean minority stress score was 14.41, indicating moderate-to-severe minority stress. A one-standard-deviation increase in minority stress was associated with higher odds of precarious employment [adjusted odds ratio (OR_adj_) 1.17, 95% confidence interval (CI) 1.08–1.26], unemployment [OR_adj_ 1.36 (95% CI 1.19–1.56)], earning ≤$20 000 USD [OR_adj_ 1.57 (95% CI 1.36-1.80)], and earning US$20 001–50 000 [OR_adj_ 1.48 (95% CI 1.32–1.66)]. Employment discrimination was reported by 14.4% of all workers and was associated with precarious employment [OR_adj_ 1.25 (95% CI 1.01–1.54)], unemployment [OR_adj_ 2.11 (95% CI 1.54–2.89)], and earning US$20 001–50 000 compared to ≥US$100 001 [OR_adj_ 1.45 (95% CI 1.07–1.96)]. Transgender and gender-diverse (TGD) workers faced poorer job quality, greater minority stress, and employment discrimination than cisgender sexual minority workers.

**Conclusions:**

Higher minority stress levels and employment discrimination were associated with worse job quality among SGM workers. Future labor market reforms should reduce minority stress and employment discrimination, as well as improve job quality, for SGM workers.

Employment is a key determinant of population health (1). Low-quality employment and unemployment are associated with poor psychosocial and health outcomes including reduced social networks, economic insecurity, and worse mental health (2). Sexual and gender minority (SGM; including but not limited to lesbian, gay, bisexual, transgender, queer, intersex, and asexual) people experience poorer employment outcomes compared to cisgender, heterosexual people – disproportionate rates of involuntary part-time employment, underemployment, unemployment, precarious employment, and lower incomes (3–5). Transgender and gender diverse (TGD) people (whose current gender identity [or identities] is not consistent with that commonly associated with their sex assigned at birth) face particularly hostile social attitudes that result in exclusion from employment and greater rates of violence, discrimination, and poverty (5, 6). These inequities persist despite anti-discrimination protections intended to safeguard SGM people from employment discrimination, such as Title VII of the United States (US) Civil Rights Act of 1964. Inequities are tied to structural (eg, government policies), institutional (eg, workplace culture and policies), and interpersonal labor market and workplace discrimination (7). Over 7% (~8 million) of the US working-age population identify as SGM people, a number that continues to grow steadily each year (8). More attention to employment is needed, particularly given employment’s impact on health and the mental health inequities that SGM populations face (9).

Minority stress – the heightened stress that sexual, gender, racial, and other minorities face due to their everyday encounters with stigma, prejudice, and discrimination – is a dominant explanation for health inequities among SGM populations (10–12). Minority stress in the workplace, including cisheteronormativity (privileging cisgender and heterosexual identities as the social norm; can lead to cisheterosexism, which is oppression targeted at people who are not both cisgender and heterosexual), impacts mental health and employment outcomes for SGM workers (13–15). Minority stress operates through distal stressors, such as external events (eg, discrimination, rejection), and proximal stressors, such as internal processes (eg, internalized stigma, concealing identity), which might shape the employment into which SGM people opt or are forced. Stigma and prejudice also shape SGM people’s relationship to the labor market through educational attainment, socioeconomic resources, and limited available opportunities (16). Previous studies on minority stress and employment have largely overlooked TGD workers, focused on cisgender sexual minority men, and emphasized workplace minority stress rather than a broader conception of minority stress on employment outcomes (13, 16, 17).

Contemporary labor markets continue to experience significant changes to job quality due to a redistribution of power that prioritizes capital accumulation over worker security and well-being (18). This has resulted in the deterioration of job quality, where the standard employment relationship (ie, full-time permanent employment) has been replaced with more flexible labor, such as non-standard (ie, little-to-no job security; highly flexible hours) and precarious employment (ie, a form of non-standard employment characterized by inadequate income and limited rights and protections) (19, 20). Due to limited gender identity and sexual orientation variables in population-level labor and occupational health surveys, SGM workers are often not considered in precarious employment and occupational health research.

With increasing hostility towards SGM populations in the US and globally (eg, anti-SGM legislation, murder of transgender women) (21) along with gender-based segregation and discrimination in the labor market, understanding how minority stress and employment discrimination shape SGM people’s lives and livelihoods is needed. This study sought to illuminate employment disparities SGM people face by exploring the associations between (i) minority stress and employment discrimination with job quality and (ii) minority stress and employment discrimination with job quality by gender. We hypothesized that greater overall minority stress and employment discrimination will be associated with lower job quality and that TGD groups will experience the poorest outcomes.

## Methods

### Study design and participants

We conducted a pooled cross-sectional analysis using 2021 (July 2022–May 2023) and 2022 (June 2022–May 2023) annual questionnaire data from The PRIDE [Population Research in Identities and Disparities for Equality] Study, an online study of SGM adults in the US. Participant recruitment occurred through a community-engaged framework (22) that utilized online advertising, community and health organizations, and SGM events across the US (23). To enroll in The PRIDE Study, participants must be 18 years or older, identify as an SGM person, live in the US, and read and understand English. For this study, we included workers – those who are either employed or unemployed but actively seeking employment – and excluded those not in the labor market (ie, unemployed and not looking for employment; homemaker; unable to work due to a disability; or retired), students, or those who are self-employed (24); data about these excluded groups are presented in the supplementary material (XXX) figure S1 and table S1. Participant consent was obtained electronically, and institutional review board/ethics approval was overseen by the Stanford University Institutional Review Board (IRB), and the WIRB-Copernicus Group (WCG) IRB for all ongoing analyses.

### Measures

*Gender groups.* Participants indicated their current gender identity from the following list (multiple selections permitted): agender, cisgender man, cisgender woman, genderqueer, man, nonbinary, questioning, transgender man, transgender woman, Two-Spirit, woman, and another gender identity. Participants also indicated their sex assigned at birth (female, male). Using a two-step process (25, 26), we cross-classified gender identity and sex assigned at birth to categorize participants into six mutually exclusive gender groups: (i) cisgender men, (ii) cisgender women, (iii) gender diverse people assigned female at birth (AFAB), (iv) gender diverse people assigned male at birth (AMAB), (v) transgender men, and (vi) transgender women (group breakdowns in supplementary table S2) (22). Write-in responses were placed into appropriately corresponding groups.

*Exposures.* We used the sexual minority stress subscale from the Cultural Assessment for the Risk of Suicide (CARS) to measure sexual minority stress among cisgender sexual minority populations (27) and an adapted subscale for gender minority stress that uses the same sexual minority stress subscale with adapted language for gender minority populations (Bryant-Lees KB et al. Minority stress model measurement: examining the validity and factor structure with sexual and gender identity inclusive revisions. Submitted). Participants selected if they wanted a questionnaire for sexual or gender minority individuals or both. They received the minority stress subscale(s) based on their selection. Each subscale consisted of five items with six Likert-type response options (ranging from 1 = strongly disagree to 6 = strongly agree). Items in both subscales include one statement related to distal stressors (ie, external events) and four statements related to proximal stressors (ie, internal thoughts and feelings). An example of one item is “The decision to hide or reveal my sexual orientation to others causes me significant distress.” (see https://pridestudy.tools/qms for full list of items). All five responses in each subscale were summed to create a continuous variable ranging from 5–30; higher scores indicated more minority stress. A score of ≥14 is indicative of moderate-severe minority stress, whereas a score of <14 is indicative of none-mild minority stress. For those who completed the sexual *and* gender minority subscales, the subscale with the higher score was used for analysis. Employment discrimination in the past 12 months (hereafter employment discrimination) was a binary variable based on: “In the PAST 12 MONTHS, have you been treated unfairly at work or when applying/interviewing for a job?”

*Outcomes*. To capture key dimensions of job quality, we considered employment type and income. *Employment type* was identified through multiple response options: ‘employed, working ≥40 hours per week,’ ‘employed, working 1–39 hours per week,’ ‘temporarily employed,’ and ‘not employed, looking for employment’ (hereafter, unemployed). Participants were categorized into three employment type groups: (i) standard employment if they selected ‘employed, working ≥40 hours per week’ only (ie, did not select ‘temporarily employed’); (ii) precarious employment if they selected ‘employed, working 1–39 hours per week,’ ‘temporarily employed,’ and/or multiple selections (eg, working ≥40 hours per week and temporarily employed); and (iii) unemployed if they selected this option *only*. We categorized temporary and part-time (ie, <40 hours per week) workers as working in precarious employment given that these forms of employment often relate to insecurity, inadequate incomes, and limited workplace protections (19). *Annual individual income* (hereafter income) was determined based on responses to: “What were your individual earnings (in US dollars) before taxes and deductions from ALL sources in the [2020, 2021] tax year?” Responses were categorized into four income categories: $0–20 000, $20 001–50 000, $50 001–100 000, and ≥$100 001.

*Socio-demographics*. Participants self-reported their ethnoracial identity, gender identity, sexual orientation, intersex identity, immigrant status, and education level. We then categorized participants ethnoracial identity into eight mutually exclusive groups: Asian; Black, African, or African American; Indigenous (‘American Indian or Alaska Native’ or ‘Native Hawaiian and other Pacific Islander’); Latino, Hispanic, or Spanish; Middle Eastern or North African; White; multiracial; and participants who selected another ethnoracial identity. Urbanicity and US Census region were assessed at the US ZIP code (postal code) level. Urbanicity was determined based on the US Department of Agriculture 2010 Rural-Urban Community Area (RUCA) codes. Urban areas included all metropolitan area primary codes (1–3) and non-metropolitan areas in which ≥30% commuted to a nearby metropolitan core [4.1, 5.1, 7.1, 8.1, and 10.1 (28)]. All other RUCA codes were categorized as rural areas. To assign US census regions, ZIP codes were aggregated based on their corresponding state-level census region classification.

### Statistical analysis

Descriptive statistics were calculated for demographic characteristics and the levels of minority stress and employment discrimination by job quality indicators. We used multinomial logistic regression to examine the associations of minority stress and employment discrimination with employment type and annual income. The multinomial regression reference categories were standard employment and income ≥$100 001, indicators of good quality employment. For each outcome, we estimated two models: an unadjusted model and a model adjusted for age, ethnoracial groups, education, immigration status, US census region, and urbanicity. Robust standard errors were estimated using the Huber-White sandwich estimator. Missingness due to non-response for potential confounders ranged from 0.8–13.3%. Multiple imputations via chained equations using the *mice* package were implemented to handle missingness with 20 imputations incorporated in all analyses (29). We conducted secondary analyses that stratified our models by gender groups, as cisheteronormativity may differentially influence how minority stress and employment discrimination are associated with job quality indicators. Results are presented as odds ratios (OR) and associated 95% confidence intervals (CI).

We conducted two sensitivity analyses. First, as part-time and temporary employment may produce differing experiences of precarity, we ran models to understand the relationship of minority stress and employment discrimination with these outcomes separately. Second, we calculated the E-value and its lower bound for adjusted models in the overall sample and by gender groups to assess unmeasured confounding. The E-value represents the minimum strength of association that an unmeasured confounder must have with both the exposure and outcome to fully explain the observed association. Since OR were calculated for common outcomes, we approximated the E-value by replacing the risk ratio with the square root of the OR (30). All analyses were conducted in R version 4.3.

## Results

The sample consisted of 4221 participants with a mean age of 37.6 years [standard deviation (SD) 12.2; Table 1]. Most participants self-identified exclusively as White (82.4%), US-born (83.7%), and currently living in an urban setting (92.5%). Participants were diverse with respect to gender identity, sexual orientation, and US census region. Regarding employment type, 71.2% of workers were in standard employment, 22.0% in precarious employment, and 6.8% were unemployed; 17.5% had incomes of ≤$20 000, and 30.7% earned $20 001–50 000. Employment discrimination was reported by 14.2% of workers. The sample mean minority stress score was 14.41 [standard deviation (SD) 5.36]. Transgender men and gender diverse AFAB people had the highest proportion of workers in precarious employment (26.5% each), followed by gender diverse workers AMAB (25.0%), cisgender women (21.9%), transgender women (19.8%), and cisgender men (15.2%). A higher proportion of transgender men and women reported unemployment (11.0% and 8.2%, respectively) than cisgender men and women (5.2% and 4.6%, respectively). Transgender men had the highest proportion of workers with income ≤$20 000 (28.3%), and cisgender men had the lowest proportion (7.9%). TGD participants had the highest minority stress scores (mean 16.44–17.75), while cisgender men and women had the lowest (mean 11.83 and 12.04, respectively). Employment discrimination was at approximately double among TGD workers compared to cisgender workers with cisgender men reporting the lowest (7.6%) and gender diverse AMAB workers the highest (21.9%).

**Table 1a:** Socio-demographic characteristics of sexual and gender minority participants in labor market, Pride Study], 2021–2023. [AFAB=assigned female at birth; AMAB=assigned male at birth.]

		Total (N=4221)		Cisgender men (N=1027)		Cisgender women (N=1162)		Gender diverse AFAB (N=1060)		Gender diverse AMAB (N=160)		Transgender men (N=555)		Transgender women (N=257)
		N (%)		N (%)		N (%)		N (%)		N (%)		N (%)		N (%)
Gender identity ^a^														
	Agender	232 (5.5)		0 (0.0)		0 (0.0)		176 (16.6)		27 (16.9)		23 (4.1)		6 (2.3)
	Cisgender man	745 (17.6)		722 (70.3)		0 (0.0)		0 (0.0)		22 (13.8)		1 (0.2)		0 (0.0)
	Cisgender woman	1056 (25.0)		0 (0.0)		944 (81.2)		108 (10.2)		2 (1.2)		2 (0.4)		0 (0.0)
	Genderqueer	606 (14.4)		0 (0.0)		0 (0.0)		421 (39.7)		64 (40.0)		93 (16.8)		28 (10.9)
	Man	803 (19.0)		520 (50.6)		0 (0.0)		7 (0.7)		35 (21.9)		236 (42.5)		5 (1.9)
	Non-binary	1097 (26.0)		0 (0.0)		0 (0.0)		759 (71.6)		108 (67.5)		169 (30.5)		61 (23.7)
	Questioning	214 (5.1)		0 (0.0)		0 (0.0)		167 (15.8)		24 (15.0)		15 (2.7)		8 (3.1)
	Transgender man	534 (12.7)		0 (0.0)		0 (0.0)		0 (0.0)		0 (0.0)		534 (96.2)		0 (0.0)
	Transgender woman	247 (5.9)		0 (0.0)		0 (0.0)		0 (0.0)		0 (0.0)		1 (0.2)		246 (95.7)
	Two-spirit	32 (0.8)		0 (0.0)		0 (0.0)		19 (1.8)		4 (2.5)		5 (0.9)		4 (1.6)
	Woman	861 (20.4)		0 (0.0)		514 (44.2)		228 (21.5)		2 (1.2)		5 (0.9)		112 (43.6)
	Another gender identity	280 (6.6)		0 (0.0)		0 (0.0)		210 (19.8)		25 (15.6)		37 (6.7)		8 (3.1)
	Missing	0 (0.0)		0 (0.0)		0 (0.0)		0 (0.0)		0 (0.0)		0 (0.0)		0 (0.0)
Intersex														
	No	4144 (98.2)		1025 (99.8)		1159 (99.7)		1022 (96.4)		154 (96.2)		540 (97.3)		244 (94.9)
	Yes	74 (1.8)		2 (0.2)		3 (0.3)		36 (3.4)		6 (3.8)		15 (2.7)		12 (4.7)
	Missing	3 (0.1)		0 (0.0)		0 (0.0)		2 (0.2)		0 (0.0)		0 (0.0)		1 (0.4)
Sexual orientation ^a^														
	Asexual	439 (10.4)		17 (1.7)		91 (7.8)		198 (18.7)		26 (16.2)		72 (13.0)		35 (13.6)
	Bisexual	1297 (30.7)		111 (10.8)		432 (37.2)		403 (38.0)		42 (26.2)		218 (39.3)		91 (35.4)
	Gay	1427 (33.8)		911 (88.7)		156 (13.4)		127 (12.0)		72 (45.0)		146 (26.3)		15 (5.8)
	Lesbian	964 (22.8)		0 (0.0)		576 (49.6)		233 (22.0)		6 (3.8)		10 (1.8)		139 (54.1)
	Pansexual	682 (16.2)		33 (3.2)		178 (15.3)		230 (21.7)		40 (25.0)		126 (22.7)		75 (29.2)
	Queer	1954 (46.3)		150 (14.6)		541 (46.6)		728 (68.7)		93 (58.1)		344 (62.0)		98 (38.1)
	Questioning	96 (2.3)		5 (0.5)		11 (0.9)		33 (3.1)		7 (4.4)		24 (4.3)		16 (6.2)
	Same-gender loving	188 (4.5)		33 (3.2)		50 (4.3)		41 (3.9)		14 (8.8)		28 (5.0)		22 (8.6)
	Heterosexual	65 (1.5)		3 (0.3)		5 (0.4)		4 (0.4)		4 (2.5)		39 (7.0)		10 (3.9)
	Two-spirit	21 (0.5)		1 (0.1)		1 (0.1)		10 (0.9)		4 (2.5)		3 (0.5)		2 (0.8)
	Another sexual orientation	150 (3.6)		10 (1.0)		26 (2.2)		76 (7.2)		11 (6.9)		15 (2.7)		12 (4.7)
	Missing	0 (0.0)		0 (0.0)		0 (0.0)		0 (0.0)		0 (0.0)		0 (0.0)		0 (0.0)
Ethnoracial identity ^a^														
	American Indian or Alaska Native	100 (2.4)		14 (1.4)		18 (1.5)		38 (3.6)		8 (5.0)		15 (2.7)		7 (2.7)
	Asian	197 (4.7)		56 (5.5)		38 (3.3)		51 (4.8)		16 (10.0)		22 (4.0)		14 (5.4)
	Black, African American or African	168 (4.0)		39 (3.8)		46 (4.0)		45 (4.2)		8 (5.0)		25 (4.5)		5 (1.9)
	Latino, Hispanic or Spanish	256 (6.1)		86 (8.4)		56 (4.8)		63 (5.9)		10 (6.2)		28 (5.0)		13 (5.1)
	Middle Eastern or North African	52 (1.2)		11 (1.1)		7 (0.6)		17 (1.6)		4 (2.5)		8 (1.4)		5 (1.9)
	Native Hawaiian or other Pacific Islander	12 (0.3)		5 (0.5)		4 (0.3)		2 (0.2)		1 (0.6)		0 (0.0)		0 (0.0)
	White	3852 (91.3)		896 (87.2)		1082 (93.1)		976 (92.1)		143 (89.4)		514 (92.6)		241 (93.8)
	Another race or ethnicity	53 (1.3)		7 (0.7)		10 (0.9)		22 (2.1)		6 (3.8)		5 (0.9)		3 (1.2)
	Missing	1 (0.0)		0 (0.0)		0 (0.0)		1 (0.1)		0 (0.0)		0 (0.0)		0 (0.0)
Ethnoracial groups														
	Asian	116 (2.7)		41 (4.0)		24 (2.1)		25 (2.4)		8 (5.0)		11 (2.0)		7 (2.7)
	Black, African American or African	95 (2.3)		29 (2.8)		26 (2.2)		20 (1.9)		1 (0.6)		16 (2.9)		3 (1.2)
	Indigenous	12 (0.3)		4 (0.4)		4 (0.3)		3 (0.3)		0 (0.0)		1 (0.2)		0 (0.0)
	Latino, Hispanic or Spanish	256 (6.1)		86 (8.4)		56 (4.8)		63 (5.9)		10 (6.2)		28 (5.0)		13 (5.1)
	Middle Eastern or North African	10 (0.2)		4 (0.4)		2 (0.2)		3 (0.3)		0 (0.0)		1 (0.2)		0 (0.0)
	White	3478 (82.4)		825 (80.3)		1001 (86.1)		854 (80.6)		123 (76.9)		458 (82.5)		217 (84.4)
	Multiracial	240 (5.7)		36 (3.5)		47 (4.0)		86 (8.1)		16 (10.0)		38 (6.8)		17 (6.6)
	Selected another identity	13 (0.3)		2 (0.2)		2 (0.2)		5 (0.5)		2 (1.2)		2 (0.4)		0 (0.0)
	Missing	1 (0.0)		0 (0.0)		0 (0.0)		1 (0.1)		0 (0.0)		0 (0.0)		0 (0.0)
Immigrant status														
	Non-US born	126 (3.0)		52 (5.1)		31 (2.7)		18 (1.7)		6 (3.8)		9 (1.6)		10 (3.9)
	US born	3533 (83.7)		854 (83.2)		983 (84.6)		902 (85.1)		127 (79.4)		463 (83.4)		204 (79.4)
	Missing	562 (13.3)		121 (11.8)		148 (12.7)		140 (13.2)		27 (16.9)		83 (15.0)		43 (16.7)
Region														
	Northeast	903 (21.4)		186 (18.1)		277 (23.8)		241 (22.7)		35 (21.9)		120 (21.6)		44 (17.1)
	Midwest	870 (20.6)		170 (16.6)		244 (21.0)		237 (22.4)		34 (21.2)		124 (22.3)		61 (23.7)
	South	1082 (25.6)		330 (32.1)		292 (25.1)		215 (20.3)		35 (21.9)		133 (24.0)		77 (30.0)
	West	1338 (31.7)		337 (32.8)		341 (29.3)		360 (34.0)		55 (34.4)		172 (31.0)		73 (28.4)
	Missing	4 (0.1)		0 (0.0)		2 (0.2)		0 (0.0)		0 (0.0)		2 (0.4)		0 (0.0)
Urbanicity														
	Rural	287 (6.8)		54 (5.3)		79 (6.8)		67 (6.3)		14 (8.8)		55 (9.9)		18 (7.0)
	Urban	3906 (92.5)		969 (94.4)		1075 (92.5)		986 (93.0)		145 (90.6)		494 (89.0)		237 (92.2)
	Missing	4 (0.1)		0 (0.0)		2 (0.2)		0 (0.0)		0 (0.0)		2 (0.4)		0 (0.0)
Survey completion year														
	2021	3108 (73.6)		782 (76.1)		866 (74.5)		780 (73.6)		113 (70.6)		384 (69.2)		183 (71.2)
	2022	1113 (26.4)		245 (23.9)		296 (25.5)		280 (26.4)		47 (29.4)		171 (30.8)		74 (28.8)
Education level														
	High school or less	139 (3.3)		26 (2.5)		20 (1.7)		28 (2.6)		3 (1.9)		46 (8.3)		16 (6.2)
	Some college	621 (14.7)		134 (13.0)		110 (9.5)		144 (13.6)		34 (21.2)		129 (23.2)		70 (27.2)
	4-year degree	1549 (36.7)		317 (30.9)		370 (31.8)		479 (45.2)		59 (36.9)		210 (37.8)		114 (44.4)
	Graduate degree	1912 (45.3)		550 (53.6)		662 (57.0)		409 (38.6)		64 (40.0)		170 (30.6)		57 (22.2)
	Missing	0 (0.0)		0 (0.0)		0 (0.0)		0 (0.0)		0 (0.0)		0 (0.0)		0 (0.0)
Employment type														
	Standard	3007 (71.2)		818 (79.6)		855 (73.6)		697 (65.8)		105 (65.6)		347 (62.5)		185 (72.0)
	Precarious	929 (22.0)		156 (15.2)		254 (21.9)		281 (26.5)		40 (25.0)		147 (26.5)		51 (19.8)
	Unemployed	285 (6.8)		53 (5.2)		53 (4.6)		82 (7.7)		15 (9.4)		61 (11.0)		21 (8.2)
Annual individual income														
	$0–20 000	738 (17.5)		81 (7.9)		149 (12.8)		263 (24.8)		34 (21.2)		157 (28.3)		54 (21.0)
	$20 001–50 000	1294 (30.7)		212 (20.6)		344 (29.6)		395 (37.3)		58 (36.2)		217 (39.1)		68 (26.5)
	$50 001–100 000	1364 (32.3)		361 (35.2)		458 (39.4)		313 (29.5)		41 (25.6)		127 (22.9)		64 (24.9)
	≥$100 001	825 (19.5)		373 (36.3)		211 (18.2)		89 (8.4)		27 (16.9)		54 (9.7)		71 (27.6)
Employment discrimination, past 12 months														
	No	3612 (85.6)		949 (92.4)		1039 (89.4)		845 (79.7)		125 (78.1)		445 (80.2)		209 (81.3)
	Yes	609 (14.4)		78 (7.6)		123 (10.6)		215 (20.3)		35 (21.9)		110 (19.8)		48 (18.7)

^a^ Participants had the option to select more than one option; thus, proportions may sum to more than 100%. Of the total sample, 9.7% selected more than one ethnoracial identity, 43.0% selected more than one gender identity, 46.6% selected more than one sexual orientation.

**Table 1b:** Socio-demographic characteristics of sexual and gender minority participants in labor market, Pride Study 2021–2023. [CARS=cultural assessment of risk of suicide; SD=standard deviation].

	Total (N=4221)		Cisgender men (N=1027)		Cisgender women (N=1162)		Gender diverse AFAB (N=1060)		Gender diverse AMAB (N=160)		Transgender men (N=555)		Transgender women (N=257)
	Mean (SD)		Mean (SD)		Mean (SD)		Mean (SD)		Mean (SD)		Mean (SD)		Mean (SD)
Age, years	37.6 (12.2)		44.6 (13.1)		37.8 (11.6)		32.4 (8.7)		36.1 (12.0)		32.7 (9.9)		42.1 (13.2)
CARS score for minority stress	14.41 (5.36)		11.83 (4.68)		12.04 (4.52)		16.75 (4.74)		16.44 (5.12)		17.75 (4.78)		17.23 (5.01)

Workers with higher minority stress scores reported poorer job quality: less favorable employment types and lower incomes. Workers who reported employment discrimination more frequently reported incomes ≤$50 000 (table 2).

**Table 2:** Minority stress and employment discrimination by job quality indicators (ie, employment type, annual individual income) among sexual and gender minority participants in the labor market, [Redacted], 2021–2023 (N=4221) [CARS=cultural assessment of risk of suicide; SD=standard deviation].

		CARS score for minority stress		Employment discrimination		
		Mean (SD)		No (N=3612)		Yes (N=609)
				N (%)		N (%)
Employment type						
	Standard	14.0 (5.3)		2620 (72.5)		387 (63.6)
	Precarious	15.1 (5.4)		779 (21.6)		150 (24.6)
	Unemployed	16.5 (5.6)		213 (5.9)		72 (11.8)
Annual individual income						
	$0–20 000	16.4 (5.3)		597 (16.5)		141 (23.2)
	$20 001–50 000	15.3 (5.2)		1076 (29.8)		218 (35.8)
	$50 001–100 000	13.6 (5.1)		1211 (33.5)		153 (25.1)
	≥$100 001	12.5 (5.2)		728 (20.2)		97 (15.9)

In adjusted models, we observed that higher minority stress scores and employment discrimination were associated with greater odds of poorer job quality (table 3). With standard employment and income ≥$100 001 as reference groups, a 1 SD increase in minority stress score was associated with higher odds of precarious employment [adjusted odds ratio (OR_adj_) 1.17; 95% CI 1.08–1.26], unemployment (OR_adj_ 1.36; 95% CI 1.19–1.56), income of ≤$20 000 (OR_adj_ 1.57; 95% CI 1.36–1.80), and income of $20 001–50 000 (OR_adj_ 1.48; 95% CI 1.32–1.66). Similarly, employment discrimination was associated with precarious employment (OR_adj_ 1.25; 95% CI 1.01–1.54), unemployment (OR_adj_ 2.11; 95% CI 1.54–2.89), and income of $20 001–50 000 (OR_adj_ 1.45; 95% CI 1.07–1.96).

**Table 3:** Associations between minority stress and employment discrimination with job quality indicators (ie, employment type, annual individual income) among sexual and gender minority participants in the labor market, Pride Study 2021–2023 (N=4221). [OR=odds ratio; CI=confidence interval].

		CARS score for minority stress			Employment discrimination	
		Unadjusted OR (95% CI)	Adjusted ^a^ OR (95% CI)		Unadjusted OR (95% CI)	Adjusted ^a^ OR (95% CI)
Employment type						
	Precarious versus standard	1.23 (1.14–1.33)	1.17 (1.08–1.26)		1.30 (1.06–1.60)	1.25 (1.01–1.54)
	Unemployed versus standard	1.59 (1.40–1.81)	1.36 (1.19–1.56)		2.29 (1.72–3.05)	2.11 (1.54–2.89)
Annual individual income						
	$0–20 000 versus ≥$100 001	2.14 (1.91–2.39)	1.57 (1.36–1.80)		1.77 (1.34–2.35)	1.44 (0.99–2.11)
	$20 001–50 000 versus ≥$100 001	1.79 (1.62–1.99)	1.48 (1.32–1.66)		1.52 (1.18–1.97)	1.45 (1.07–1.96)
	$50 001–100 000 versus ≥$100 001	1.27 (1.16–1.40)	1.20 (1.08–1.32)		0.95 (0.72–1.24)	0.90 (0.68–1.20)

^a^ Adjusted estimates are controlled for age, education level, ethnoracial group, immigrant status, US Census region, urbanicity, and survey completion year.

Minority stress scores were typically highest among workers reporting unemployment and incomes $0–50 000 (table 4). Transgender men reported the highest mean minority stress scores, including by unemployment (19.4; SD 4.7) and income of ≤$20 000 (19.2; SD 5.0). Transgender women and gender diverse AFAB and AMAB workers had similar scores among these job quality indicators. Compared to those who did not experience employment discrimination, those who did had a higher percentage of being unemployed. Employment discrimination by income levels differed between cisgender and TGD workers. Among those who reported employment discrimination, a large proportion of TGD workers reported having a lower income ($0–50 000), while a smaller proportion of TGD workers reported this with higher incomes (>$50 000). Among cisgender workers, the inverse was true.

**Table 4:** Minority stress and employment discrimination by job quality indicators and gender groups among sexual- and gender-minority participants in the labor market, [Redacted], 2021–2023 (N=4221) [AFAB=assigned female at birth; AMAB=assigned male at birth; CARS=cultural assessment of risk of suicide; SD= standard deviation].

Gender group outcomes per employment type and income			CARS score for minority stress	Employment discrimination	
				No	Yes
			Mean (SD)	N (%)	N (%)
**Cisgender men (N=1027)**					
	Standard employment		11.6 (4.5)	772 (81.3)	46 (59.0)
	Precarious employment		12.5 (4.8)	139 (14.6)	17 (21.8)
	Unemployed		13.2 (5.9)	38 (4.0)	15 (19.2)
	Annual income				
		$0–20 000	12.4 (4.6)	69 (7.3)	12 (15.4)
		$20 001–50 000	13.0 (4.9)	194 (20.4)	18 (23.1)
		$50 001–100 000	11.8 (4.7)	338 (35.6)	23 (29.5)
		≥$100 001	11.0 (4.5)	348 (36.7)	25 (32.1)
**Cisgender women (N=1162)**					
	Standard employment		11.9 (4.5)	763 (73.4)	92 (74.8)
	Precarious employment		12.2 (4.7)	233 (22.4)	21 (17.1)
	Unemployed		13.3 (4.4)	43 (4.1)	10 (8.1)
	Annual income				
		$0–20 000	12.8 (4.5)	137 (13.2)	12 (9.8)
		$20 001–50 000	12.7 (4.9)	304 (29.3)	40 (32.5)
		$50 001–100 000	11.8 (4.3)	415 (39.9)	43 (35.0)
		≥$100 001	10.9 (4.2)	183 (17.6)	28 (22.8)
**Gender diverse AFAB (N=1060)**					
	Standard employment		16.6 (4.8)	558 (66.0)	139 (64.7)
	Precarious employment		16.8 (4.4)	222 (26.3)	59 (27.4)
	Unemployed		18.0 (4.7)	65 (7.7)	17 (7.9)
	Annual income				
		$0–20 000	17.6 (4.6)	213 (25.2)	50 (23.3)
		$20 001–50 000	17.1 (4.5)	303 (35.9)	92 (42.8)
		$50 001–100 000	16.1 (4.8)	260 (30.8)	53 (24.7)
		≥$100 001	15.0 (5.4)	69 (8.2)	20 (9.3)
**Gender diverse AMAB (N=160)**					
	Standard employment		15.8 (4.7)	87 (69.6)	18 (51.4)
	Precarious employment		17.4 (5.6)	30 (24.0)	10 (28.6)
	Unemployed		18.6 (6.2)	8 (6.4)	7 (20.0)
	Annual income				
		$0–20 000	18.8 (4.8)	23 (18.4)	11 (31.4)
		$20 001–50 000	16.1 (4.7)	44 (35.2)	14 (40.0)
		$50 001–100 000	16.4 (5.0)	36 (28.8)	5 (14.3)
		≥$100 001	14.3 (5.7)	22 (17.6)	5 (14.3)
**Transgender men (N=555)**					
	Standard employment		17.3 (4.5)	283 (63.6)	64 (58.2)
	Precarious employment		18.1 (5.3)	118 (26.5)	29 (26.4)
	Unemployed		19.4 (4.7)	44 (9.9)	17 (15.5)
	Annual income				
		$0–20 000	19.2 (5.0)	117 (26.3)	40 (36.4)
		$20 001–50 000	17.6 (4.7)	173 (38.9)	44 (40.0)
		$50 001–100 000	16.7 (4.4)	108 (24.3)	19 (17.3)
		≥$100 001	16.9 (4.3)	47 (10.6)	7 (6.4)
**Transgender women (N=257)**					
	Standard employment		17.2 (5.2)	157 (75.1)	28 (58.3)
	Precarious employment		17.4 (4.2)	37 (17.7)	14 (29.2)
	Unemployed		16.9 (4.8)	15 (7.2)	6 (12.5)
	Annual income				
		$0–20 000	17.3 (4.6)	38 (18.2)	16 (33.3)
		$20 001–50 000	17.8 (5.0)	58 (27.8)	10 (20.8)
		$50 001–100 000	16.6 (5.1)	54 (25.8)	10 (20.8)
		≥$100 001	17.2 (5.3)	59 (28.2)	12 (25.0)

Associations of minority stress with job quality were similar among gender groups with the strongest associations among cisgender men and the weakest associations among transgender women (table 5). A higher minority stress score was positively and significantly associated with unemployment among cisgender men (OR_adj_ 1.46; 95% CI 1.02–2.07) only. Higher minority stress scores were positively and significantly associated with incomes ≤$20 000 among cisgender men (OR_adj_ 1.25; 95% CI 1.05–1.50), gender diverse AMAB workers (OR_adj_ 2.79; 95% CI 1.13–6.89), and transgender men (OR_adj_ 2.63; 95% CI 1.35–5.14). Higher minority stress scores were also positively and significantly associated with incomes of $20 001–50 000 for cisgender men (OR_adj_ 1.95; 95% CI 1.25–2.03) and cisgender women (OR_adj_ 1.49; 95% CI 1.14–1.96) but not TGD people. Associations between employment discrimination and job type varied across gender groups and were not significant with the exception of precariously employed (OR_adj_ 2.11; 95% CI 1.09–4.09) and unemployed (OR_adj_ 6.94; 95% CI 3.14–15.3) cisgender men. Last, there was little association with employment discrimination and income, except among cisgender men with incomes ≤$20 000 (OR_adj_ 2.30; 95% CI 1.00–5.27).

**Table 5:** Associations between minority stress and employment discrimination with job quality indicators (ie, employment type, annual individual income) by gender groups among sexual and gender minority participants in the labor market, Pride Study, 2021–2023 (N=4221). [AFAB=assigned female at birth; AMAB=assigned male at birth; CARS=cultural assessment of risk of suicide; [OR=odds ratio; CI=confidence interval].

Gender group outcomes per employment type and income		CARS score for minority stress	Employment discrimination
		Adjusted ^a^ OR (95% CI)	Adjusted ^a^ OR (95% CI)
**Cisgender men (N=1027)**			
Employment type			
	Precarious versus standard	1.22 (0.99–1.49)	2.11 (1.09–4.09)
	Unemployed versus standard	1.46 (1.02–2.07)	6.94 (3.14–15.3)
Annual income			
	$0–20 000 versus ≥$100 001	1.25 (1.05–1.50)	2.30 (1.00–5.27)
	$20 001–50 000 versus ≥$100 001	1.59 (1.25–2.03)	1.18 (0.59–2.37)
	$50 001–100 000 versus ≥$100 001	1.21 (1.01–1.46)	0.96 (0.51–1.81)
**Cisgender women (N=1162)**			
Employment type			
	Precarious versus standard	1.04 (0.87–1.25)	0.70 (0.40–1.20)
	Unemployed versus standard	1.24 (0.91–1.69)	1.59 (0.72–3.52)
Annual income			
	$0–20 000 versus ≥$100 001	1.40 (0.94–2.09)	0.57 (0.28–1.19)
	$20 001–50 000 versus ≥$100 001	1.49 (1.14–1.96)	1.11 (0.55–2.23)
	$50 001–100 000 versus ≥$100 001	1.24 (0.98–1.57)	0.71 (0.40–1.28)
**Gender diverse AFAB (N=1060)**			
Employment type			
	Precarious versus standard	0.97 (0.82–1.15)	1.02 (0.72–1.46)
	Unemployed versus standard	1.21 (0.88–1.65)	1.03 (0.53–2.00)
Annual income			
	$0–20 000 versus ≥$100 001	1.40 (0.94–2.10)	0.82 (0.38–1.79)
	$20 001–50 000 versus ≥$100 001	1.39 (0.99–1.94)	1.21 (0.62–2.33)
	$50 001–100 000 versus ≥$100 001	1.27 (0.95–1.70)	0.74 (0.38–1.43)
**Gender diverse AMAB (N=160)**			
Employment type			
	Precarious versus standard	1.41 (0.90–2.22)	1.15 (0.41–3.20)
	Unemployed versus standard	2.13 (0.97–4.67)	4.09 (0.89–18.8)
Annual income			
	$0–20 000 versus ≥$100 001	2.79 (1.13–6.89)	2.41 (0.49–11.9)
	$20 001–50 000 versus ≥$100 001	1.28 (0.70–2.37)	0.74 (0.34–6.25)
	$50 001–100 000 versus ≥$100 001	1.47 (0.80–2.70)	0.42 (0.10–2.10)
**Transgender men (N=555)**			
Employment type			
	Precarious versus standard	1.07 (0.83–1.38)	0.93 (0.55–1.58)
	Unemployed versus standard	1.50 (0.92v2.43)	1.53 (0.72–3.27)
Annual income			
	$0–20 000 versus ≥$100 001	2.63 (1.35–5.14)	2.03 (0.49–8.67)
	$20 001–50 000 versus ≥$100 001	0.94 (0.62–1.43)	2.25 (0.55–9.18)
	$50 001–100 000 versus ≥$100 001	0.86 (0.55–1.35)	1.17 (0.37–3.70)
**Transgender women (N=257)**			
Employment type			
	Precarious versus standard	1.01 (0.72–1.43)	2.09 (0.96–4.54)
	Unemployed versus standard	0.84 (0.55–1.26)	2.30 (0.79–6.69)
Annual income			
	$0–20 000 versus ≥$100 001	0.84 (0.49–1.44)	2.70 (0.83–8.84)
	$20 001–50 000 versus ≥$100 001	1.20 (0.78–1.86)	0.98 (0.35–2.79)
	$50 001–100 000 versus ≥$100 001	0.91 (0.62–1.34)	0.79 (0.29–2.15)

^a^ Controlled for age, education level, ethnoracial group, immigrant status, US census region, and urbanicity. P-value for interaction for job quality and minority stress = 0.204 P-value for interaction for income and minority stress < 0.001 P-value for interaction for job quality and discrimination = 0.043 P-value for interaction for income and discrimination = 0.420

Further sensitivity analyses that stratified precarious workers into part-time and temporary workers found that minority stress scores were typically higher among temporary workers than part-time workers (supplemental table S3), and the association of minority stress and employment discrimination was stronger with temporary employment for the overall sample and for cisgender men, gender diverse AFAB workers, and gender diverse AMAB workers (supplementary table S4). The E-value for minority stress and employment discrimination among the overall sample ranged from 1.38–2.26 (supplementary table S5). For example, an unmeasured confounder would need to be related to minority stress and precarious employment with a magnitude of ≥ 1.38 beyond the measured covariates to fully explain away the results.

## Discussion

Our study extends the literature on SGM people in the labor market by assessing job quality rather than employment status (employed *versus* unemployed), identifying an association between minority stress and employment discrimination with job quality, and highlighting differences by gender. TGD workers reported the highest minority stress, most employment discrimination, and worst job quality compared to cisgender workers. Previous studies exploring the relationship between minority stress and employment have focused on workplace and job satisfaction (13, 17) rather than broader labor market outcomes such as employment type and income. Our study highlights the important role of cisheternormativity for SGM people in the labor market. The precarious employment group in our study was heterogenous and included temporary and part-time workers. Sensitivity analyses demonstrated that associations of minority stress and discrimination with precarious employment could be driven primarily by temporary employment and less by part-time employment.

Few studies address job quality among SGM populations. In past research, sexual minority people (presumably cisgender) were approximately three times as likely to experience precarious (as opposed to standard) employment relative to their heterosexual counterparts (4). Our study included TGD people, who have largely been overlooked in socioeconomic and labor market research (3). Existing literature identified poorer employment outcomes among TGD workers than cisgender workers. Our study expands this and demonstrates poorer outcomes compared to cisgender sexual minority workers as well (5, 31). We demonstrated substantially more precarious employment, unemployment, and lower incomes among TGD workers compared to cisgender sexual minority workers, who have already been identified as facing significantly poorer job quality than heterosexual people (4).

Though the mean minority stress score of 14.4 indicated moderate or severe minority stress (27) among many participants, scores differed greatly by gender. TGD groups scored at least four points higher than cisgender sexual minority groups. Similar patterns were observed for employment discrimination with TGD people reporting approximately two or more times the prevalence compared to cisgender sexual minorities. Discrimination is an important characteristic of precarious employment (32). The prevalence of employment discrimination and its association with job quality in our findings demonstrates the importance of studying discrimination in employment-related research among SGM workers (8).

Nearly half (48.2%) of our sample earned <$50 001, the approximate average income (~$50 310) of all US adults in the 2023 census; this was 61.3% among TGD workers in our study (33). Unemployment among TGD people in our sample is over double the US population unemployment rate (~4%) in 2021–2022 (34). There is limited US based population-level data on SGM employment to compare with our study. Our results were similar to those in the 2015 US Transgender Survey (USTS). In our study, a smaller proportion of transgender men (11.0%) and transgender women (8.2%) were unemployed compared to 12.6% and 12.2%, respectively, in the 2015 USTS (35). Combining all employed workers included and excluded from our study (standard, precarious, self-employed, and employed students; supplementary figure S1) to provide comparable proportions, our sample had higher proportions of employment (71.5% of transgender men and 69.4% of transgender women) than those in the USTS (68.5% of transgender men and 67% of transgender women) (35).

Our study highlights that considering a binary employment status alone – as often considered in the literature – is insufficient to understand the diverse labor market outcomes of SGM people. For example, transgender women in our study and elsewhere (35) appear to have poorer outcomes (eg, higher unemployment) compared to transgender men by employed *versus* unemployed comparisons, but transgender women appear to have similar or better job quality than transgender men in our study. This is further nuanced by the finding that transgender men, along with gender diverse AMAB and AFAB people, were more likely to be out of the labor market because of a disability (41.0%, 63.5%, and 37.6%, respectively) compared to transgender women, cisgender men, and cisgender women (25.8%, 27.5% and 24.8%, respectively) (supplementary table S1). Noteworthy, age can contribute to differences in employment outcomes, such as employment quality improving with age. Transgender women in our sample were older, and a higher proportion were retired, compared to transgender men.

The association of minority stress scores and job quality varied marginally by gender group, demonstrating that minority stress may not differentially shape job quality significantly by gender. However, the association of employment discrimination and job quality did vary by gender group. Cisgender men on average had some of the strongest associations, with cisgender women and gender diverse AFAB workers having the weakest. Previous studies highlighted that employment discrimination can impact job satisfaction, job commitment, and health (8). The pathways by which minority stress and employment discrimination interact with job quality may be different between TGD and cisgender workers and should be further explored.

### Cisheteronormativity, minority stress, and employment discrimination

Cisheteronormativity leads to a status quo in which SGM people encounter everyday hostility and discrimination leading to minority stress (36, 37). Minority stress processes that stem from cisheteronormativity are demonstrated in the high minority stress scores across our sample (27). Some cisgender heterosexual people may feel uncomfortable or negatively toward SGM communities, with TGD people viewed particularly unfavorably, impacting labor market inclusion (38). Further, through distal and proximal stressors, minority stress can impact the careers and sectors (eg, arts, service industry) that some SGM workers may enter to reduce exposure to discrimination (some lower quality jobs may provide safety, acceptance, and workplace satisfaction) (6). These stressors also negatively impact SGM workers’ mental health that can impact their labor market integration (39). Furthermore, these careers, sectors, or challenges integrating into the labor market may lead to increased employment precarity. Employment discrimination may also shape the types of jobs that SGM workers can access or choose to go into for reasons of safety (6). This may explain the associations identified between minority stress and job quality indicators in our study. Non-cisheteronormative ways of being (eg, gender non-binary expression) may be viewed as unprofessional or misunderstood in cisheteronormative spaces, including workplaces (40). This can occur by being required to adhere to binary gender norms related to dress, mannerisms, or language (41) or through hiring biases for stereotypically more masculine or feminine jobs (42). This leads to discrimination among SGM people looking for employment and on the job with approximately half of SGM people having experienced unfair treatment at work (8, 42, 43). In the past 12 months, 14.4% of our sample experienced employment discrimination with a greater proportion reported by TGD workers.

### Strengths and limitations

To our knowledge, this is the largest SGM employment study, enabling the characterization of employment outcomes among six gender groups. The inclusion of employment-related variables beyond binary comparisons of employment status is a strength that will contribute to the existing literature on job quality among SGM populations. However, the results should be interpreted considering several limitations.

We used a modified CARS subscale to evaluate SGM-related minority stress, and items from the CARS scale may be interpreted differently across gender groups. Since the original CARS scale was validated primarily in a cisgender sample (27), our adaptation of the CARS scale may not fully capture certain latent constructs that are important to minority stress in TGD individuals. Further, the measure used here to capture minority stress was chosen for its brevity and because of its demonstrated relationship with important minority stress related correlates (mental health and biological outcomes) (27, 44) but does not capture all minority stress experiences related to employment. The limited ethnoracial diversity of the sample and focus on minority stress based on sexual orientation and gender identity means that we did not fully capture the discrimination and mistreatment of ethno-racial SGM workers. Future research might consider increased nuance of how minority stress may operate similarly and differently for cisgender sexual minority people and gender minority people of any sexual orientation, as well as those who experience other systemic oppressions (eg, racism, biphobia).

Our measures of job quality and precarious employment did not consider subjective measures (eg, job satisfaction) or the multidimensional nature (ie, consisting of limited job security, inadequate income, and limited rights and protections) of precarious employment that will be beneficial in future studies for a more fulsome understanding of job quality (19). Missing information on potential confounders (eg, occupation, industry) is a limitation; however, our sensitivity analysis suggested that the overall sample estimate is robust to these unmeasured confounders. The cross-sectional study design allowed for the possibility that participants may have experienced poorer job quality, and this might have influenced how they reported on the CARS scale or experiences of employment discrimination. Finally, this study used a convenience sample, which may limit the generalizability of the findings and may be subjected to sampling and recall biases.

### Interventions

Interventions should aim to reduce minority stress and employment discrimination while improving job quality among SGM workers until research can disentangle the relationships between job quality, minority stress, and employment discrimination. Unfortunately, cisheteronormativity operates well beyond the labor market and shapes many life domains (11). Addressing cisheteronormativity, which undergirds minority stress and discrimination in the labor market, may lead to improved employment outcomes for SGM people (7). Likewise, higher quality jobs may be associated with SGM-inclusive policies and workplace culture, which reduces the minority stress and employment discrimination experienced by SGM workers. Providing improved access (*eg*, SGM mentorship programs) for SGM people to these high-quality jobs may reduce minority stress and employment discrimination.

Attention to the sociostructural conditions (eg, cisheteronormativity) that produce minority stress and permit employment discrimination is needed (11, 13). Tangible, effective interventions to improve employment outcomes among SGM people at structural and workplace levels have been identified. SGM people living in jurisdictions with anti-discrimination and human rights laws (eg, same-gender marriage) have significantly better labor market outcomes (7). The political ideology of jurisdictions (eg, liberal state governors and legislatures) and less anti-SGM attitudes result in better employment outcomes for SGM people. At the workplace level, policies related to anti-discrimination and inclusion of SGM workers (diversity policies and enacted measures – eg, all-gender restrooms) improve employment experiences for SGM people (7). Many US states and workplaces lack SGM anti-discrimination and human rights policies, a clear area for immediate intervention to reduce minority stress and employment discrimination among SGM people and improve employment outcomes. These interventions will not just benefit SGM workers in the US but also those excluded from the labor market by increasing safety when looking for employment and on the job.

## Supplementary material

Supplementary material

## References

[1] Landsbergis PA, Choi B, Dobson M, Sembajwe G, Slatin C, Delp L et al. The key role of work in population health inequities. Am J Public Health 2018 Mar;108(3):296–7. 10.2105/AJPH.2017.304288 29412716 PMC5803831

[2] Rönnblad T, Grönholm E, Jonsson J, Koranyi I, Orellana C, Kreshpaj B et al. Precarious employment and mental health: a systematic review and meta-analysis of longitudinal studies. Scand J Work Environ Health 2019 Sep;45(5):429–43. 10.5271/sjweh.3797 31165899

[3] Badgett L. The Economic Case for LGBT Equality: Why Fair and Equal Treatment Benefits Us All. Beacon Press; 2020.

[4] Kinitz DJ, Shahidi FV, Ross LE. Job quality and precarious employment among lesbian, gay, and bisexual workers: A national study. SSM Popul Health 2023 Oct;24:101535. 10.1016/j.ssmph.2023.101535 38021458 PMC10661442

[5] Waite S, Ecker J, Ross LE. A systematic review and thematic synthesis of Canada’s LGBTQ2S+ employment, labour market and earnings literature. PLoS One 2019 Oct;14(10):e0223372. 10.1371/journal.pone.0223372 31577824 PMC6774499

[6] Kinitz DJ, Shahidi FV, Kia H, MacKinnon K, MacEachen E, Gesink D et al. Precarious Employment: A Neglected Issue Among Lesbian, Gay, Bisexual, and Transgender Workers. Sex Res Soc Policy 2024;•••:1–17. 10.1007/s13178-024-00950-3

[7] Gould WA, Kinitz DJ, Shahidi FV, MacEachen E, Mitchell C, Venturi DC et al. Improving LGBT labor market outcomes through laws, workplace policies, and support programs: A scoping review. Sex Res Soc Policy 2024;•••:1–18. 10.1007/s13178-023-00918-9

[8] Sears B, Castleberry NM, Lin A, Mallory C. LGBTQ People’s Experiences of Workplace Discrimination and Harassment 2023. Williams Inst UCLA [Internet]. 2024 Aug 27 [cited 2025 Feb 6]; Available from: https://escholarship.org/content/qt45c9q04k/qt45c9q04k.pdf

[9] Kidd SA, Howison M, Pilling M, Ross LE, McKenzie K. Severe mental illness in LGBT populations: A scoping review. Psychiatr Serv 2016 Jul;67(7):779–83. 10.1176/appi.ps.201500209 26927576 PMC4936529

[10] Brooks VR. Minority stress and lesbian women. Free Press; 1981.

[11] Meyer IH. Prejudice, social stress, and mental health in lesbian, gay, and bisexual populations: conceptual issues and research evidence. Psychol Bull 2003 Sep;129(5):674–97. 10.1037/0033-2909.129.5.674 12956539 PMC2072932

[12] Hendricks ML, Testa RJ. A conceptual framework for clinical work with transgender and gender nonconforming clients: An adaptation of the Minority Stress Model. Prof Psychol Res Pr 2012 Oct;43(5):460–7. 10.1037/a0029597

[13] Waldo CR. Working in a majority context: A structural model of heterosexism as minority stress in the workplace. J Couns Psychol 1999;46(2):218. 10.1037/0022-0167.46.2.218

[14] Ross LE, Kinitz DJ, Kia H. Pronouns Are a Public Health Issue. Am J Public Health 2022 Mar;112(3):360–2. 10.2105/AJPH.2021.306678 35196057 PMC8887152

[15] Perales F, Ablaza C, Elkin N. Exposure to Inclusive Language and Well-Being at Work Among Transgender Employees in Australia, 2020. Am J Public Health 2022 Mar;112(3):482–90. 10.2105/AJPH.2021.306602 35196034 PMC8887154

[16] Kinitz DJ, Ross LE, MacEachen E, Fehr C, Gesink D. "…full of opportunities, but not for everyone": A narrative inquiry into mechanisms of labor market inequity among precariously employed gay, bisexual, and queer men. Am J Ind Med 2024 Apr;67(4):350–63. 10.1002/ajim.23574 38374777

[17] Holman EG. Theoretical extensions of minority stress theory for sexual minority individuals in the workplace: A cross‐contextual understanding of minority stress processes. J Fam Theory Rev 2018 Mar;10(1):165–80. 10.1111/jftr.12246 30008806 PMC6040646

[18] Benach J, Vives A, Amable M, Vanroelen C, Tarafa G, Muntaner C. Precarious employment: understanding an emerging social determinant of health. Annu Rev Public Health 2014;35(1):229–53. 10.1146/annurev-publhealth-032013-182500 24641559

[19] Kreshpaj B, Orellana C, Burström B, Davis L, Hemmingsson T, Johansson G et al. What is precarious employment? A systematic review of definitions and operationalizations from quantitative and qualitative studies. Scand J Work Environ Health 2020 May;46(3):235–47. 10.5271/sjweh.3875 31901944

[20] International Labour Organization. Non-standard employment around the world: understanding challenges, shaping prospects [Internet]. ILO Geneva; 2016. Available from: https://www.ilo.org/wcmsp5/groups/public/---dgreports/---dcomm/---publ/documents/publication/wcms_534326.pdf

[21] Westbrook L. The matrix of violence: intersectionality and necropolitics in the murder of transgender people in the United States, 1990–2019. Gend Soc 2023;37(3):413–46. 10.1177/08912432231171172

[22] Obedin-Maliver J, Hunt C, Flentje A, Armea-Warren C, Bahati M, Lubensky ME et al. Engaging Sexual and Gender Minority (SGM) Communities for Health Research: Building and Sustaining PRIDEnet. J Community Engagem Scholarsh 2024;16(2):9. 10.54656/jces.v16i2.484 39149568 PMC11326444

[23] Lunn MR, Lubensky M, Hunt C, Flentje A, Capriotti MR, Sooksaman C et al. A digital health research platform for community engagement, recruitment, and retention of sexual and gender minority adults in a national longitudinal cohort study--The PRIDE Study. J Am Med Inform Assoc 2019 Aug;26(8-9):737–48. 10.1093/jamia/ocz082 31162545 PMC6696499

[24] Peckham T, Flaherty B, Hajat A, Fujishiro K, Jacoby D, Seixas N. What does non-standard employment look like in the United States? An empirical typology of employment quality. Soc Indic Res 2022 Sep;163(2):555–83. 10.1007/s11205-022-02907-8 37006816 PMC10062421

[25] Bauer GR, Braimoh J, Scheim AI, Dharma C. Transgender-inclusive measures of sex/gender for population surveys: mixed-methods evaluation and recommendations. PLoS One 2017 May;12(5):e0178043. 10.1371/journal.pone.0178043 28542498 PMC5444783

[26] Tate CC, Ledbetter JN, Youssef CP. A two-question method for assessing gender categories in the social and medical sciences. J Sex Res 2013;50(8):767–76. 10.1080/00224499.2012.690110 22989000

[27] Chu J, Floyd R, Diep H, Pardo S, Goldblum P, Bongar B. A tool for the culturally competent assessment of suicide: the Cultural Assessment of Risk for Suicide (CARS) measure. Psychol Assess 2013 Jun;25(2):424–34. 10.1037/a0031264 23356681

[28] Rural Health Research Centre. RUCA Data [Internet]. University of Washington; n.d. [cited 2024 May 14]. Available from: https://depts.washington.edu/uwruca/ruca-uses.php

[29] Van Buuren S, Groothuis-Oudshoorn K. mice: multivariate imputation by chained equations in R. J Stat Softw 2011;45:1–67. 10.18637/jss.v045.i03

[30] VanderWeele TJ, Ding P. Sensitivity Analysis in Observational Research: introducing the E-Value. Ann Intern Med 2017 Aug;167(4):268–74. 10.7326/M16-2607 28693043

[31] Ciprikis K, Cassells D, Berrill J. Transgender labour market outcomes: evidence from the United States. Gend Work Organ 2020;27(6):1378–401. 10.1111/gwao.12501

[32] Vives A, Amable M, Ferrer M, Moncada S, Llorens C, Muntaner C et al. The Employment Precariousness Scale (EPRES): psychometric properties of a new tool for epidemiological studies among waged and salaried workers. Occup Environ Med 2010 Aug;67(8):548–55. 10.1136/oem.2009.048967 20576923

[33] Guzman G, Kollar M. Income in the United States: 2023 [Internet]. Washington, DC: United States Census Bureau; 2024 Sep [cited 2025 Jan 19] p. 1–59. (Current Population Reports). Available from: https://www2.census.gov/library/publications/2024/demo/p60-282.pdf

[34] Bureau of Labor Statistics [Internet]. [cited 2024 Jun 10]. Civilian unemployment rate. Available from: https://www.bls.gov/charts/employment-situation/civilian-unemployment-rate.htm

[35] Leppel K. Transgender men and women in 2015: Employed, unemployed, or not in the labor force. J Homosex 2021 Jan;68(2):203–29. 10.1080/00918369.2019.1648081 31403900

[36] Chevrette R, Eguchi S. We Don’t See LGBTQ Differences”: Cisheteronormativity and Concealing Phobias and Irrational Fears Behind Rhetorics of Acceptance. QED J GLBTQ Worldmaking. 2020;7(1):55–9. 10.14321/qed.7.1.0055

[37] Frost DM, Meyer IH. Minority stress theory: Application, critique, and continued relevance. Curr Opin Psychol 2023 Jun;51:101579. 10.1016/j.copsyc.2023.101579 37270877 PMC10712335

[38] Lewis DC, Flores AR, Haider-Markel DP, Miller PR, Tadlock BL, Taylor JK. Degrees of acceptance: variation in public attitudes toward segments of the LGBT community. Polit Res Q 2017;70(4):861–75. 10.1177/1065912917717352

[39] Kinitz DJ, Ross LE, MacEachen E, Gesink D. ‘How can you worry about employment and survival at the same time?’: employment and mental health among precariously employed cisgender and transgender sexual minority adult men in Toronto, Canada. Cult Health Sex 2024 Sep;0(0):1–16. 10.1080/13691058.2024.2408349 39342499

[40] Kinitz D, Gould WA, Shahidi F, MacEachen E, Mitchell C, Craig-Venturi D et al. Addressing knowledge gaps about skills of 2SLGBTQ+ people in Canada: A scoping review and qualitative inquiry. A report for Employment and Social Development Canada [Internet]. University of Toronto; 2023 Jan. Available from: https://lgbtqhealth.ca/projects/docs/skillstrainingandemployment.pdf

[41] Mills S, Owens B. Customer Abuse and Aggression as Labour Control Among LGBT Workers in Low-Wage Services. Work Employ Soc [Internet]. 2021; Available from: https://www.scopus.com/inward/record.uri?eid=2-s2.0-85118513157&doi=10.1177%2f09500170211045843&partnerID=40&md5=bafb77ba02ed3148cb3b6943e7501f1a 10.1177/09500170211045843 PMC10230591 37265819

[42] Dilmaghani M, Robinson M. The blue of the rainbow: queerness and hiring discrimination in blue-collar occupations. Rev Soc Econ 2022;•••:1–29.

[43] Waite S. Should I stay or should I go? Employment discrimination and workplace harassment against transgender and other minority employees in Canada’s federal public service. J Homosex 2021 Sep;68(11):1833–59. 10.1080/00918369.2020.1712140 31951793

[44] Ghanooni D, Carrico AW, Williams R, Glynn TR, Moskowitz JT, Pahwa S et al. Sexual Minority Stress and Cellular Aging in Methamphetamine-Using Sexual Minority Men With Treated HIV. Psychosom Med 2022 Oct;84(8):949–56. 10.1097/PSY.0000000000001123 35980781 PMC9553259

