# Supplementary material for: Associations of minority stress and employment discrimination with job quality among sexual- and gender-minority workers
Source: Scand J Work Environ Health. 2025 Apr 27;51(3):214–25. doi: 10.5271/sjweh.4221 (PMC12071185; doi:10.5271/sjweh.4221)
Supplement: Supplementary material [file SJWEH-51-214-S001.docx]

Associations of minority stress and employment discrimination with job quality among sexual- and gender-minority workers^1^

*by David J Kinitz, PhD,  ^2^ Nguyen K Tran, PhD, Faraz Vahid Shahidi, PhD, Joelle T Maslak, BA, Annesa Flentje, PhD, Micah E Lubensky, PhD, Juno Obedin-Maliver, MD, Mitchell R Lunn, MD*

1. Supplementary material
2. Correspondence to: David J. Kinitz, PhD, Department of Medicine, Stanford University School of Medicine, 3180 Porter Drive, Palo Alto, CA 94304, USA. [E-mail: djkinitz@stanford.edu]

**Supplementary Figure S1.** Labor force status by gender groups, [Redacted], 2021 – 2023 (n = 6553). AFAB, assigned female at birth; AMAB, assigned male at birth.

**
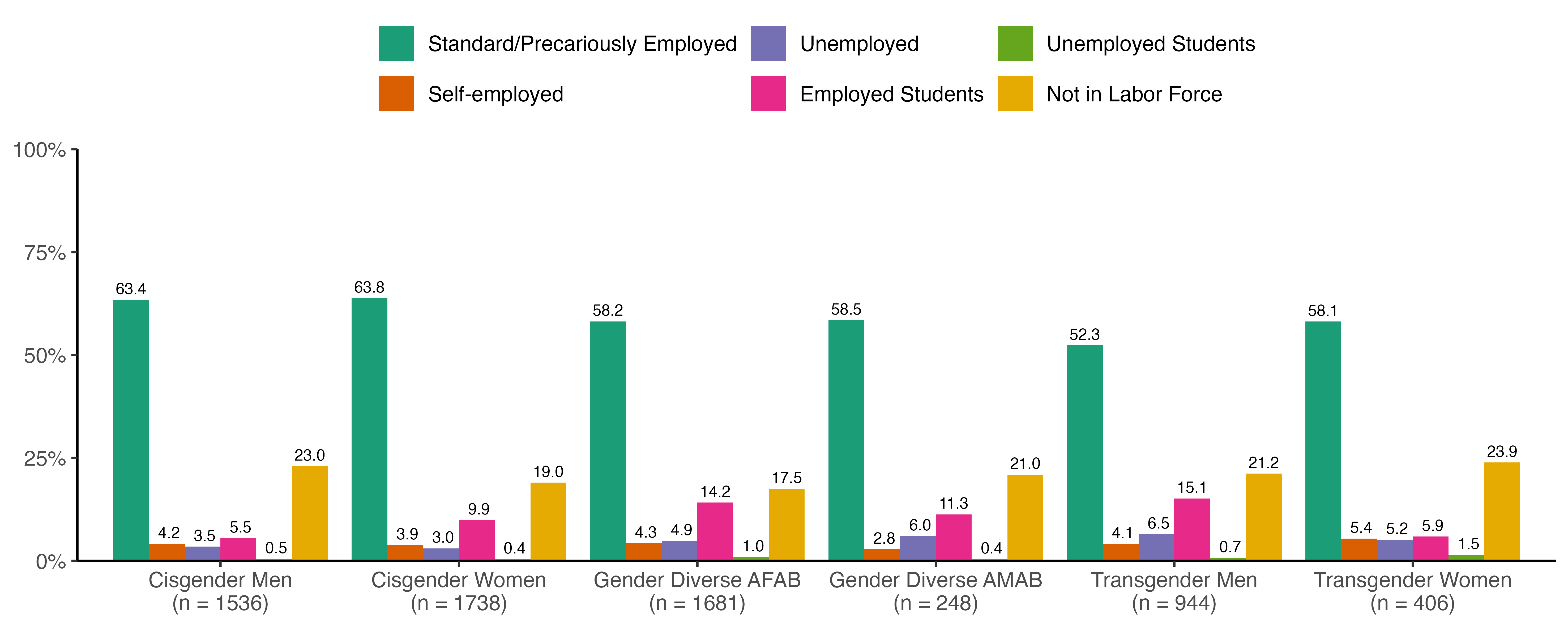
**

**Supplementary Table S1.** Minority stress and occupational status by gender groups among sexual and gender minority participants not in the labor market, [Redacted], 2021 – 2023 (n = 1327)

|  | Cisgender men  n = 353 | | Cisgender women  n = 330 | | Gender diverse AFAB  n = 295 | | Gender diverse AMAB  n = 52 | | Transgender men  n = 200 | | Transgender women  n = 97 | |
| --- | --- | --- | --- | --- | --- | --- | --- | --- | --- | --- | --- | --- |
|  | n (%) | Mean (s.d.) | n (%) | Mean (s.d.) | n (%) | Mean (s.d.) | n (%) | Mean (s.d.) | n (%) | Mean (s.d.) | n (%) | Mean (s.d.) |
| CARS score for minority stress |  | 11.4 (5.1) |  | 12.3 (5.1) |  | 17.5 (5.2) |  | 15.8 (6.5) |  | 18.0 (5.2) |  | 17.2 (4.6) |
| Occupational status^a^ |  |  |  |  |  |  |  |  |  |  |  |  |
| Unemployed, not looking for work | 50  (14.2) |  | 63  (19.1) |  | 65  (22.0) |  | 14  (26.9) |  | 38  (19.0) |  | 20  (20.6) |  |
| Homemaker | 9  (2.5) |  | 35  (10.6) |  | 19  (6.4) |  | 6  (11.5) |  | 13  (6.5) |  | 10  (10.3) |  |
| Full-time student | 41  (11.6) |  | 96  (29.1) |  | 136  (46.1) |  | 13  (25.0) |  | 80  (40.0) |  | 15  (15.5) |  |
| Part-time student | 4  (1.1) |  | 8  (2.4) |  | 11  (3.7) |  | 2  (3.8) |  | 16  (8.0) |  | 2  (2.1) |  |
| Disable, not able to work | 97  (27.5) |  | 82  (24.8) |  | 111  (37.6) |  | 19  (36.5) |  | 82  (41.0) |  | 25  (25.8) |  |
| Retired | 214  (60.6) |  | 109  (33.0) |  | 16  (5.4) |  | 12  (23.1) |  | 14  (7.0) |  | 45  (46.4) |  |

Note: CARS, Cultural Assessment of Risk of Suicide; s.d., standard deviation.

^a^ Participants had the option to select more than one option; thus, proportions may sum to more than 100%.

**Supplementary Table S2.** Descriptions of gender groups

| Gender Group | Gender identity and sex assigned at birth combinations used to categorize gender groups |
| --- | --- |
| Cisgender men | [‘cisgender man’ and/or ‘man’] *and* assigned male at birth [AMAB] |
| Cisgender women | [‘cisgender woman’ and/or ‘woman’] *and* assigned female at birth [AFAB] |
| Gender diverse people AFAB | [nonbinary or gender diverse identity, such as ‘agender,’ ‘genderqueer,’ and/or ‘another gender identity’] *and* AFAB |
| Gender diverse people AMAB | [nonbinary or gender diverse identity] *and* AMAB |
| Transgender men | ‘transgender man’ irrespective of their assigned sex at birth or reporting [‘cisgender man’ and/or ‘man’] *and* AFAB |
| Transgender women | ‘transgender woman’ irrespective of their assigned sex at birth or reporting [‘cisgender woman’ and/or ‘woman’] *and* AMAB |

**Supplementary Table S3.** Minority stress and employment discrimination by expanded employment type and gender groups among sexual and gender minority participants in the labor market, [Redacted], 2021 – 2023 (n = 4221)

|  | Employment type |  | CARS Score for Minority Stress | Employment Discrimination | |
| --- | --- | --- | --- | --- | --- |
|  |  |  |  | No | Yes |
|  |  | n (%) | Mean (s.d.) | n (%) | n (%) |
| Total (n = 4221) | Standard | 3007 (71.2) | 14.0 (5.3) | 2620 (72.5) | 387 (63.6) |
|  | Part-time | 766 (18.1) | 15.0 (5.4) | 658 (18.2) | 108 (17.7) |
|  | Temporary | 163 (3.9) | 15.7 (5.3) | 121 (3.4) | 42 (6.9) |
|  | Unemployed | 285 (6.8) | 16.5 (5.6) | 213 (5.9) | 72 (11.8) |
| Cisgender Men (n = 1027) | Standard | 818 (79.6) | 11.6 (4.5) | 772 (81.3) | 46 (59.0) |
|  | Part-time | 132 (12.9) | 12.3 (4.8) | 121 (12.8) | 11 (14.1) |
|  | Temporary | 23 (2.3) | 13.5 (5.1) | 18 (1.9) | 6 (7.7) |
|  | Unemployed | 53 (5.2) | 13.2 (5.9) | 38 (4.0) | 15 (19.2) |
| Cisgender Women (n = 1162) | Standard | 855 (73.6) | 11.9 (4.5) | 763 (73.4) | 92 (74.8) |
|  | Part-time | 213 (18.3) | 12.2 (4.8) | 198 (19.1) | 15 (12.2) |
|  | Temporary | 41 (3.5) | 12.2 (4.2) | 35 (3.4) | 6 (4.9) |
|  | Unemployed | 53 (4.6) | 13.3 (4.4) | 43 (4.1) | 10 (8.1) |
| Gender Diverse Assigned Female at Birth (n = 1060) | Standard | 697 (65.8) | 16.6 (4.8) | 558 (66.0) | 139 (64.7) |
|  | Part-time | 232 (21.9) | 16.8 (4.4) | 190 (22.5) | 42 (19.5) |
|  | Temporary | 49 (4.6) | 16.7 (4.6) | 32 (3.8) | 17 (7.9) |
|  | Unemployed | 82 (7.7) | 18.0 (4.7) | 65 (7.7) | 17 (7.9) |
| Gender Diverse Assigned Male at Birth (n = 160) | Standard | 105 (65.6) | 15.8 (4.7) | 87 (69.6) | 18 (51.4) |
|  | Part-time | 27 (16.9) | 16.6 (5.6) | 22 (17.6) | 5 (14.3) |
|  | Temporary | 13 (8.1) | 19.0 (5.4) | 8 (6.4) | 5 (14.3) |
|  | Unemployed | 15 (9.4) | 18.6 (6.2) | 8 (6.4) | 7 (20.0) |
| Transgender Men (n = 555) | Standard | 347 (62.5) | 17.3 (4.5) | 283 (63.6) | 64 (58.2) |
|  | Part-time | 122 (22.0) | 18.0 (5.4) | 98 (22.0) | 23 (21.8) |
|  | Temporary | 25 (4.5) | 18.6 (5.1) | 20 (4.5) | 5 (4.5) |
|  | Unemployed | 61 (11.0) | 19.4 (4.7) | 44 (9.9) | 17 (15.5) |
| Transgender Women (n = 257) | Standard | 185 (72.0) | 17.2 (5.2) | 157 (75.1) | 28 (58.3) |
|  | Part-time | 40 (15.6) | 17.2 (4.4) | 29 (13.9) | 11 (22.9) |
|  | Temporary | 11 (4.3) | 18.0 (3.7) | 8 (3.8) | 3 (6.2) |
|  | Unemployed | 21 (8.2) | 16.9 (4.8) | 15 (7.2) | 6 (12.5) |

Note: s.d., standard deviation.

**Supplementary Table S4**. Associations between minority stress and employment discrimination with expanded employment type by gender groups among sexual and gender minority participants in the labor market, [Redacted], 2021 – 2023 (n = 4221)

|  | Employment type | CARS Score for Minority Stress | Employment Discrimination |
| --- | --- | --- | --- |
|  |  | Adjusted OR  (95% CI) | Adjusted OR  (95% CI) |
| Total (n = 4221) | Part-time vs. Standard | 1.20 (1.11-1.31) | 1.11 (0.88-1.39) |
|  | Temporary vs. Standard | 1.37 (1.17-1.61) | 2.31 (1.59-3.34) |
|  | Unemployed vs. Standard | 1.51 (1.33-1.71) | 2.14 (1.59-2.88) |
| Cisgender Men (n = 1027) | Part-time vs. Standard | 1.15 (0.92-1.43) | 1.61 (0.74-3.50) |
|  | Temporary vs. Standard | 1.56 (0.99-2.44) | 4.78 (1.62-14.0) |
|  | Unemployed vs. Standard | 1.46 (1.02-2.07) | 6.94 (3.14-15.3) |
| Cisgender Women (n = 1162) | Part-time vs. Standard | 1.03 (0.79-1.24) | 0.58 (0.31-1.08) |
|  | Temporary vs. Standard | 1.05 (0.69-1.58) | 1.27 (0.50-3.23) |
|  | Unemployed vs. Standard | 1.24 (0.91-1.69) | 1.59 (0.72-3.52) |
| Gender Diverse Assigned Female at Birth (n = 1060) | Part-time vs. Standard | 0.98 (0.82-1.18) | 0.85 (0.57-1.27) |
|  | Temporary vs. Standard | 0.87 (0.60-1.28) | 2.17 (1.12-4.21) |
|  | Unemployed vs. Standard | 1.21 (0.88-1.65) | 1.03 (0.53-2.00) |
| Gender Diverse Assigned Male at Birth (n = 160) | Part-time vs. Standard | 1.04 (0.66-1.64) | 0.90 (0.25-3.22) |
|  | Temporary vs. Standard | 2.59 (1.15-5.84) | 1.82 (0.41-8.01) |
|  | Unemployed vs. Standard | 2.13 (0.97-4.67) | 4.09 (0.89-18.8) |
| Transgender Men (n = 555) | Part-time vs. Standard | 1.02 (0.78-1.34) | 0.92 (0.52-1.63) |
|  | Temporary vs. Standard | 1.32 (0.69-2.52) | 1.01 (0.34-2.98) |
|  | Unemployed vs. Standard | 1.50 (0.92-2.43) | 1.53 (0.72-3.27) |
| Transgender Women (n = 257) | Part-time vs. Standard | 0.97 (0.66-1.43) | 2.22 (0.94-5.27) |
|  | Temporary vs. Standard | 1.46 (0.70-3.05) | 2.59 (0.50-13.3) |
|  | Unemployed vs. Standard | 0.84 (0.55-1.26) | 2.30 (0.79-6.69) |

Note: CARS, Cultural Assessment of Risk of Suicide; CI, confidence interval; OR, odds ratio.

Adjusted estimates are controlled for age, education level, ethnoracial groups, immigrant status, U.S. Census region, urbanicity, and survey completion year.

p-value for interaction for job quality and minority stress = 0.320

p-value for interaction for job quality and discrimination = 0.053

**Supplementary Table S5.** E-values for associations between minority stress and employment discrimination with job quality indicators

| Exposure | Outcome | OR (95% CI)  reported in paper | E-value for OR (outcome prevalence >15%) | E-value for lower bound of 95% CI |
| --- | --- | --- | --- | --- |
| **Overall Sample** | | | | |
| CARS Score for Minority Stress | Precarious vs. Standard | 1.17 (1.08-1.26) | 1.38 | 1.24 |
|  | Unemployed vs. Standard | 1.36 (1.19-1.56) | 1.61 | 1.41 |
|  | $0 – $20,000 vs. ≥$100,001 | 1.57 (1.36-1.80) | 1.82 | 1.61 |
|  | $20,001 – $50,000 vs. ≥$100,001 | 1.48 (1.32-1.66) | 1.73 | 1.56 |
|  | $50,001 – $100,000 vs. ≥$100,001 | 1.20 (1.08-1.32) | 1.42 | 1.24 |
| Employment Discrimination | Precarious vs. Standard | 1.25 (1.01-1.54) | 1.48 | 1.08 |
|  | Unemployed vs. Standard | 2.11 (1.54-2.89) | 2.26 | 1.79 |
|  | $20,001 – $50,000 vs. ≥$100,001 | 1.45 (1.07-1.96) | 1.70 | 1.22 |
| **Stratified by Gender Groups** | | | | |
| *Cisgender Men* | | | | |
|  | Unemployed vs. Standard | 1.46 (1.02-2.07) | 1.71 | 1.11 |
|  | $0 – $20,000 vs. ≥$100,001 | 1.25 (1.05-1.50) | 1.48 | 1.18 |
|  | $20,001 – $50,000 vs. ≥$100,001 | 1.59 (1.25-2.03) | 1.83 | 1.48 |
|  | $50,001 – $100,000 vs. ≥$100,001 | 1.21 (1.01-1.46) | 1.43 | 1.08 |
| Employment Discrimination | Precarious vs. Standard | 2.11 (1.09-4.09) | 2.26 | 1.26 |
|  | Unemployed vs. Standard | 6.94 (3.14-15.3) | 4.71 | 2.94 |
|  | $0 – $20,000 vs. ≥$100,001 | 2.30 (1.00-5.27) | 2.40 | 1.00 |
| *Cisgender Women* | | | | |
| CARS Score for Minority Stress | $20,001 – $50,000 vs. ≥$100,001 | 1.49 (1.14-1.96) | 1.74 | 1.34 |
| *Gender Diverse Assigned Male at Birth* | | | | |
| CARS Score for Minority Stress | $0 – $20,000 vs. ≥$100,001 | 2.79 (1.13-6.89) | 2.73 | 1.32 |
| *Transgender Men* | | | | |
| CARS Score for Minority Stress | $0 – $20,000 vs. ≥$100,001 | 2.63 (1.35-5.14) | 2.63 | 1.60 |

Note: CI, confidence interval; OR, odds ratio.
